# Prognostic Value of Basic Fibroblast Growth Factor (bFGF) in Lung Cancer: A Systematic Review with Meta-Analysis

**DOI:** 10.1371/journal.pone.0147374

**Published:** 2016-01-29

**Authors:** Mingming Hu, Ying Hu, Jiabei He, Baolan Li

**Affiliations:** Department of General Medicine, Beijing Tuberculosis and Thoracic tumor research Institute/ Beijing Chest Hospital, Capital Medical University, Beijing, 101149, China; Catalan Institute of Oncology, SPAIN

## Abstract

**Background:**

Basic fibroblast growth factor (bFGF) is known to stimulate angiogenesis and thus to influence the proliferation, migration and survival of tumor cells. Many studies examined the relationship between human bFGF overexpression and survival in lung cancer patients, but the results have been mixed. To systematically summarize the clinical prognostic function of bFGF in lung cancer, we performed this systematic review with meta-analysis.

**Method:**

Studies were identified by an electronic search of PubMed, EMBASE, China National Knowledge Infrastructure and Wanfang databases, including publications prior toAugust 2014. Pooled hazard ratios (HR) for overall survival (OS) were aggregated and quantitatively analyzed by meta-analysis.

**Results:**

Twenty-two studies (n = 2154) were evaluated in the meta-analysis. Combined HR suggested that bFGF overexpression had an adverse impact on survival of patients with lung cancer(HR = 1.202,95%CI, 1.022–1.382). Our subgroup analysis revealed that the combined HR evaluating bFGF expression on OS in operable non-small cell lung cancer (NSCLC) was 1.553 (95%CI, 1.120–1.986); the combined HR in small cell lung cancer (SCLC) was 1.667 (95%CI, 1.035–2.299). There was no significant impact of bFGF expression on survival in advanced NSCLC.

**Conclusion:**

This meta-analysis showed that bFGF overexpression is a potential indicator of worse prognosis for patients with operable NSCLC and SCLC, but is not associated with outcome in advanced NSCLC. The data suggests that high bFGF expression is highly related to poor prognosis. Nevertheless,more high-quality studies should be performed in order to provide additional evidence for the prognostic value of bFGF in lung cancer.

## Introduction

Lung cancer has become a major public health problem around the world. The prognosis for lung cancer patients is poor, with an overall 5-year survival rate of approximately 15%, and little improvement has been made in recent decades [1,2]. Additionally, there is a subset of patients who have a particularly poor prognosis, even amongst those at the same stage of the disease. Non-small cell lung cancer (NSCLC) is a heterogeneous disease: its natural history is unique in each patient, as tumor-related heterogeneity, including histological and molecular features, affects treatment outcomes. There is an urgent need for reliable indicators to add prognostic information and generate personalized treatment in addition to the currently used tumor, node, metastasis (TNM) staging system.

Angiogenesis, which is the formation of new blood vessels from the endothelium of existing vasculature, plays a pivotal role in tumor growth, progression and metastasis [3]. A series of angiogenic factors are overexpressed in tumors, such as vascular endothelial growth factor (VEGF) and its receptors, fibroblast growth factor (FGF) and its receptors, hepatocyte growth factor (HGF), interleukins (ILs-1, 6, and 8) and stromal cell derived factor 1, transforming growth factorβ (TGFβ) and endothelin [4]. There are 18 mammalian FGF ligands and 4 FGF receptors (FGFR1-4) [5,6]. Basic FGF (bFGF), also known as FGF-2, is the most extensively studied peptide. bFGF is able to bind FGFR1, FGFR2, and FGFR3, leading to auto-phosphorylation of intracellular tyrosine residues, which are involved in instigating tumor cell proliferation and invasion in various tumor types [7]. The expression of MMP-1, HGF, Bcl2, survivin, MMP-9 and MMP-13 is up-regulated through bFGF and results in a gain of invasive and anti-apoptotic properties [8–11]. Deregulation of FGF signaling in tumors has been reported in various tumor types. A number of studies have explored the prognostic value of bFGF in lung cancer patients, but the results were contradictory, and therefore a consensus has not been reached. We found no meta-analysis data on the correlation of bFGF expression with survival in lung cancer patients. Thus, we decided to conduct a meta-analysis to investigate the association between bFGF overexpression and overall survival (OS) in lung cancer, so as to shed light on personalized therapy of lung cancer patients.

## Materials and Methods

### Search strategy and selection criteria

**Fig 1:**
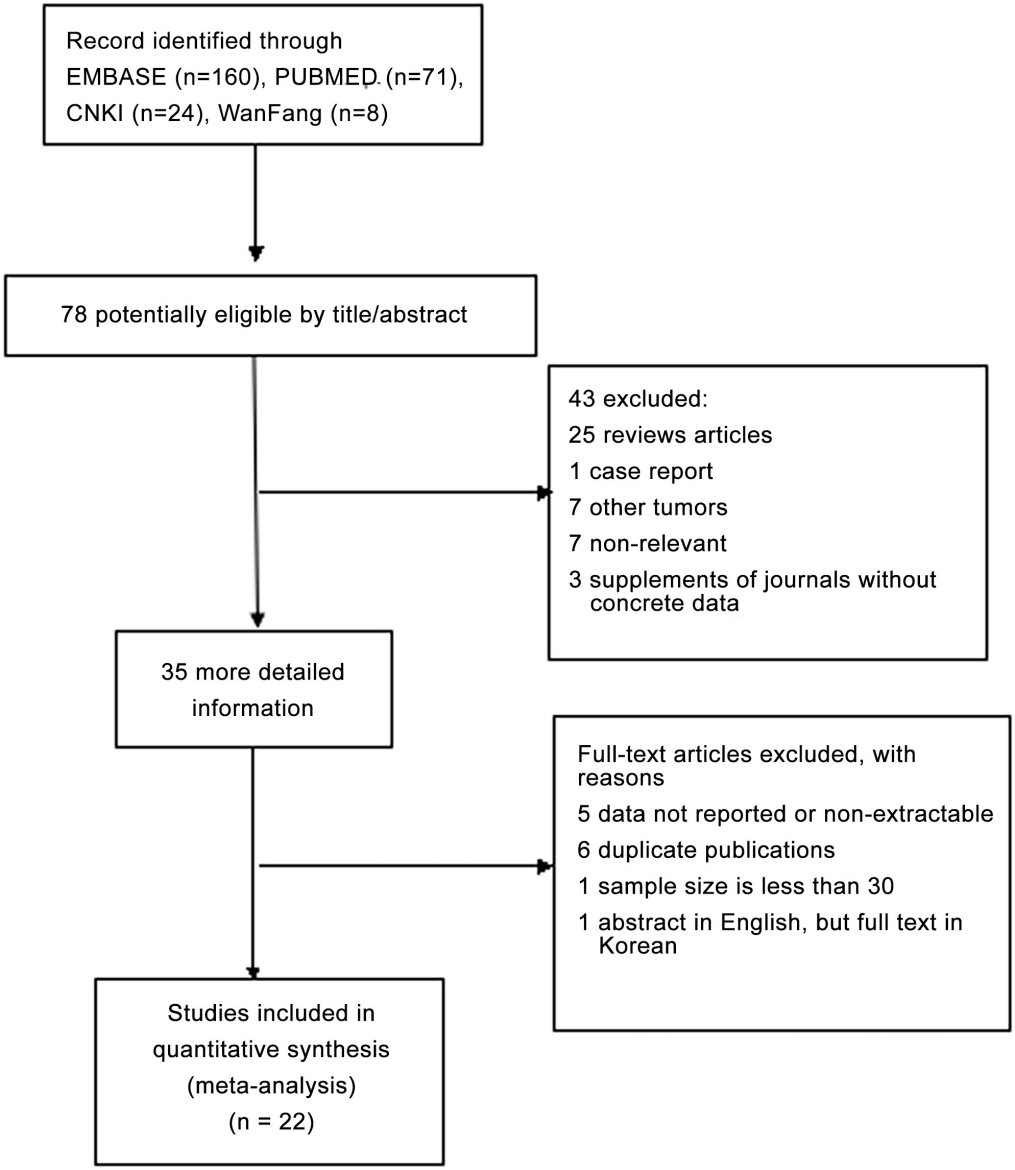
Flow chart of studies included in this meta-analysis.

We searched PubMed, EMBASE, China National Knowledge Infrastructure and Wanfang databases for relevant articles published up to August 1, 2014. Search key words included “basic fibroblast growth factor”, “bFGF”, “FGF-2”, “lung cancer”, and “prognosis or survival or outcome”. The references cited by the potentially eligible studies were also manually checked. Eligible articles were selected with the following criteria: (1) trials studied lung cancer patients; (2) association between bFGF and survival was evaluated; (3) bFGF was dichotomized as a categorical variable; (4) trials were fully published as a complete study in English or Chinese for data collection; (5) only the most complete or the recent research was included in case of multiple publications. Ultimately, 22 studies with 2154 patients were included in the meta-analysis (Fig 1).

### Data extraction and quality assessment

Each eligible article was reviewed independently by two investigators (MMH and YH). Discrepancies were recognized and fully discussed. Data was retrieved from full text, including first author, year of publication, journal of publication, patient source, the size of the cohort, study design, disease stage, histology, test method, information on the main reagents, the cutoff value, the time of follow-up and survival data. If authors reported both univariate and multivariate survival analysis results, the latter was included in our analysis. Quality assessment was performed by two investigators independently, using a quality score according to Steele’s method [12], which is the methodological scale of biologic prognostic factors used for lung cancer by the European Lung Cancer Working Party (ELCWP). Four categories are scored, including scientific design, laboratory methodology, generalizability and analysis. Final scores were expressed as percentages, with higher values indicating a better methodology.

### Statistical methods

Nonparametric tests (Mann-Whitney tests) were used to compare the distribution of quality scores according to the value of a discrete variable. For quantitative aggregation of the survival results, hazard ratio (HR) and 95% confidence intervals (CI) were used as effective values to measure the effects of bFGF overexpression on survival of lung cancer patients. For studies without published HRs or 95%CIs, we tried to contact authors requesting more details, but got no replies. Therefore, we employed a widely used method to estimate HRs and 95%CIs [13]. Statistical heterogeneity among included studies was assessed using I^2^ and the Cochran Q statistic. For the I^2^ statistic, heterogeneity was interpreted as absent (I^2^<25%), moderate (I^2^ = 25%-50%), or extreme (I^2^ = 50%-100%). For the Q statistic, p<0.10 was considered statistically significant for heterogeneity. If significant heterogeneity was denoted by a p value <0.10, random effects models were used to report HRs; otherwise, fixed-effects models were used to estimate the pooled HR when no substantial heterogeneity was observed. By convention, a pooled HR>1 implied worse survival for the group with increased bFGF expression. To validate the robustness of the meta-analysis findings, we performed sensitivity analysis by removing one study in turn. Publication bias was assessed using Begg’s test. Subgroup analysis was performed to explore the influence of histological type, disease stage and study design on outcomes. All calculations were performed using STATA version 12.0 (Stata Corporation, College Station, TX). A p<0.05 (two sided) was considered to be statistically significant.

## Results

### Eligible studies

In total, 22 studies [14–35] published between 1996 and 2014 were eligible for this meta-analysis, including NSCLC and small cell lung cancer (SCLC). The total number of patients included was 2154, ranging from 31 to 335 patients per study (mean, 98). The major characteristics of the 22 eligible publications are reported in Table 1.

**Table 1:** Main characteristics of 22 eligible studies in the meta-analysis. IHC, immunohistochemistry; ELISA, enzyme linked immunosorbent assay; AC, adenocarcinoma; SCC, squamous cell carcinoma; Non-SCC, not squamous cell carcinoma; HR, hazard ration; ED, extensive-stage disease; LD, limited-disease stage; NA, not applicable; NSCLC, non-small cell lung cancer; SCLC, small cell lung cancer.

Auhtor	Year	Ethnicity	Histology	Stage	Method	Sample size	% positive	Study design	HR(95%CI)
Takanami [14]	1996	Asian	AC	I-IV	IHC	143	NA	Retrospective	2.26(1.23–4.17)
Ito [15]	2002	Asian	AC	I-IV	IHC	80	86.3%	Retrospective	1.8(0.25–12.86)
Kojima [16]	2002	Asian	AC	I	IHC	94	64.9%	Retrospective	1.729(0.689–4.355)
Mok [17]	2014	Mixed	Non-SCC	IIIB/IV	ELISA	283	50%	Prospective	1.11(0.82–1.50)
Dowlati [18]	2008	USA	Non-SCC	IIIB/IV	ELISA	150	50%	Prospective	1.00(0.72–1.39)
Brattström [19]	1998	Europe	NSCLC	I-IV	ELISA	68	38.2%	Retrospective	0.9(0.50–1.61)
Joensuu [20]	2002	Europe	NSCLC	I-IV	ELISA	99	33.3%	Retrospective	1.6(1.0–2.70)
Garpenstrand [21]	2004	Europe	NSCLC	IIIA-IV	ELISA	33	44%	Retrospective	1.31(0.53–3.28)
Iwasaki [22]	2004	Asian	NSCLC	I-III	ELISA	71	53.5%	Retrospective	2.308(1.115–4.77)
Zhou [23]	2004	Asian	NSCLC	I-III	IHC	56	44.60%	Retrospective	3.11(1.44–6.69)
Yu [24]	2005	Asian	NSCLC	I-IV	IHC	74	NA	Retrospective	2.049(0.984–4.263)
Donnem [25]	2009	Europe	NSCLC	I-IIIA	IHC	335	8%	Retrospective	1.8(1.03–3.14)
Brattström [26]	2002	Europe	NSCLC	I-IV	ELISA	58	31%	Retrospective	0.95(0.28–3.28)
Zhao [27]	2011	Asian	NSCLC	I-IV	IHC	68	80.9%	Retrospective	1.723(0.454–6.541)
Shou [28]	2001	Asian	NSCLC	I-III	IHC	111	79.30%	Retrospective	1.00(0.53–1.89)
Kelly [29]	2011	USA	NSCLC	IV	ELISA	32	50%	Prespective	2.59(0.54–12.33)
Rades [30]	2012	Europe	NSCLC	II-III	IHC	60	43%	Retrospective	3.25(1.51–7.3)
Behrens [31]	2008	USA	SCC	I-IV	IHC	125	NA	Retrospective	0.55(0.33–0.92)
Ueno [32]	2001	Asian	SCLC	Ed+LD	ELISA	46	58.70%	Retrospective	2.5(1.169–5.348)
Ruotsalainen [33]	2002	Europe	SCLC	Ed+LD	ELISA	103	75%	Prospective	1.5(0.9–2.4)
Jiang [34]	2013	Asian	SCLC	Ed+LD	ELISA	34	52.9%	Retrospective	1.51(0.53–5.48)
Horn [35]	2009	USA	SCLC	ED	ELISA	31	50%	Prospective	2.06(0.96–4.42)

These publications followed several different patient cohorts. Among the 22 studies, 4 studies included SCLC patients, while 18 studies included NSCLC patients. The NSCLC groups contained either all lung cancer subtypes (n = 12), adenocarcinomas (n = 3), squamous cell carcinomas (SCC) (n = 1), or non-squamous carcinomas (n = 2). Twelve studies in the NSCLC group included operable NSCLC patients, while 4studiesincludedadvanced NSCLC patients. In the included studies, 10 studies used immunohistochemistry (IHC) and 12 studies used ELISA to determine bFGF expression. Five studies were designed prospectively and 17 studies were designed retrospectively. In 14 out of the 22 studies, bFGF overexpression showed no statistically significant impact on OS, an indicator of poor prognosis in 7 studies, and only 1 study identified it as an indicator of longer OS.

### Quality assessment

The overall global score ranged from 45% to 71.25%, with a mean of 57.5%. Concerning the global score, there was no statistically significant difference among 8 significant studies and 14 non-significant studies (mean of 58.9% versus 56.8%, p = 0.608). No statistically significant difference was shown between Asian and non-Asian studies according to the global score (mean of 54.4% versus 59.6% respectively, p = 0.105). Similarly, there was no difference in the examining method (ELISA versus IHC) or histology type (NSCLC versus SCLC) (mean of 58.4% versus 56.4%, p = 0.468; mean of 57.5% versus 57.4%, p = 1.00, Table 2). Due to the absence of difference between groups classified by the variables above, we performed a quantitative aggregation of all the survival results.

**Table 2:** Results of quality assessments according to ELCWP criteria. Score distributions are expressed by the mean values. IHC, immunohistochemistry; ELISA, enzyme linked immunosorbent assay; NSCLC, non-small cell lung cancer; SCLC, small cell lung cancer; Significant, significant prognostic factor for survival (P<0.05); Non-significant, not significant prognostic factor for survival (P>0.05).

	Studies(n)	Global score(%)	Design	Laboratory methodology	Generalizability	Result analysis
All studies	22	57.8	5.36	5.31	6.73	5.59
Non-Asian	11	59.6	5.55	5.48	7.0	5.82
Asian	10	54.4	5.10	5.15	6.20	5.30
P		0.105	0.213	0.76	0.247	0.310
Non-significant	14	56.8	5.43	5.0	6.79	5.5
Significant	8	58.9	5.25	5.85	6.63	5.75
P		0.608	0.653	0.036	0.726	0.672
NSCLC	18	57.5	5.39	5.33	6.67	5.61
SCLC	4	57.4	5.25	5.20	7.00	5.50
P		1.00	0.888	0.889	0.631	0.895
ELISA	12	58.4	5.42	5.28	7.17	5.50
IHC	10	56.4	5.30	5.35	6.20	5.70
P		0.468	0.800	0.885	0.156	0.865

### Meta-analysis

**Fig 2:**
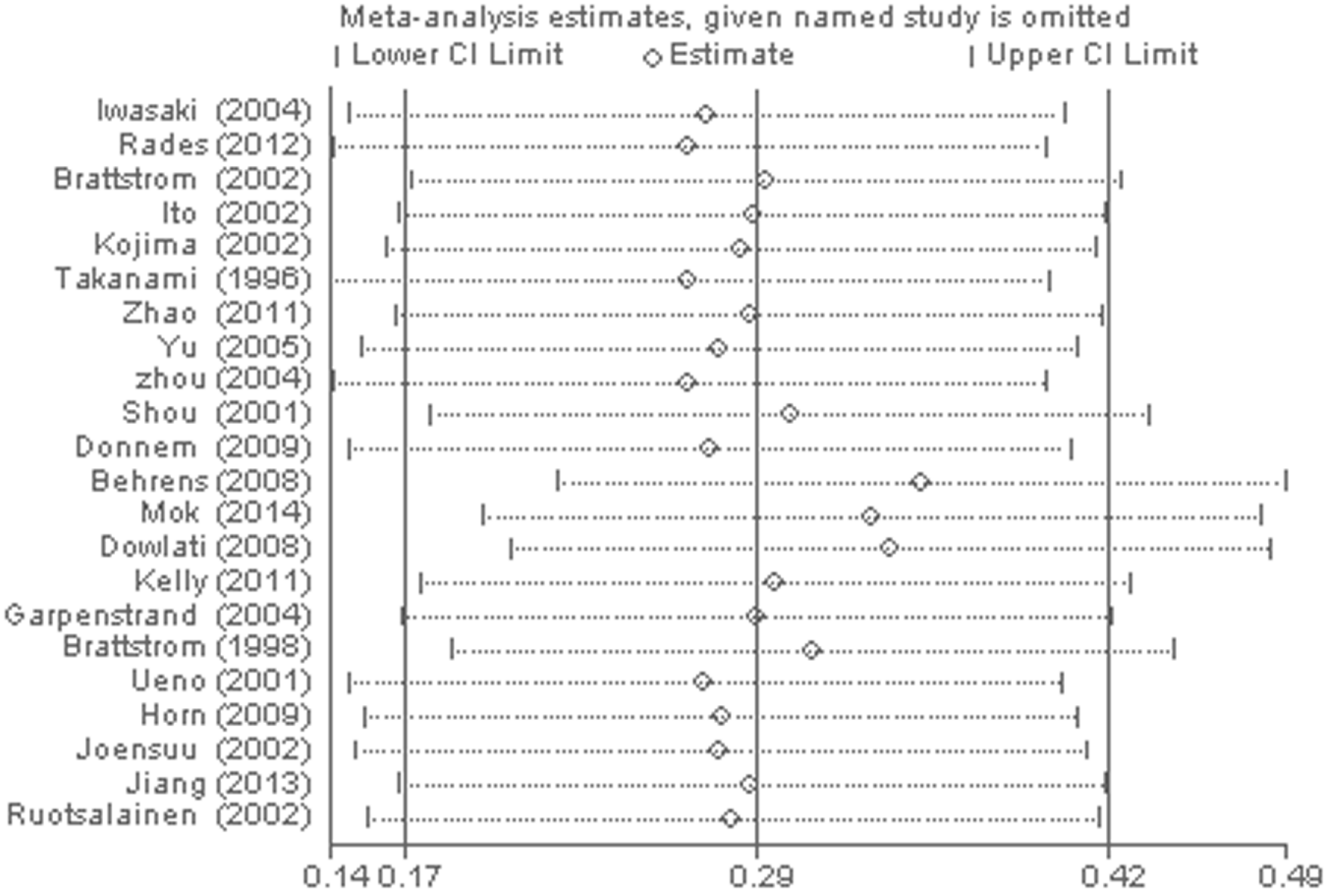
Sensitivity analysis for combined HRs evaluating bFGF expression on OS.

**Fig 3:**
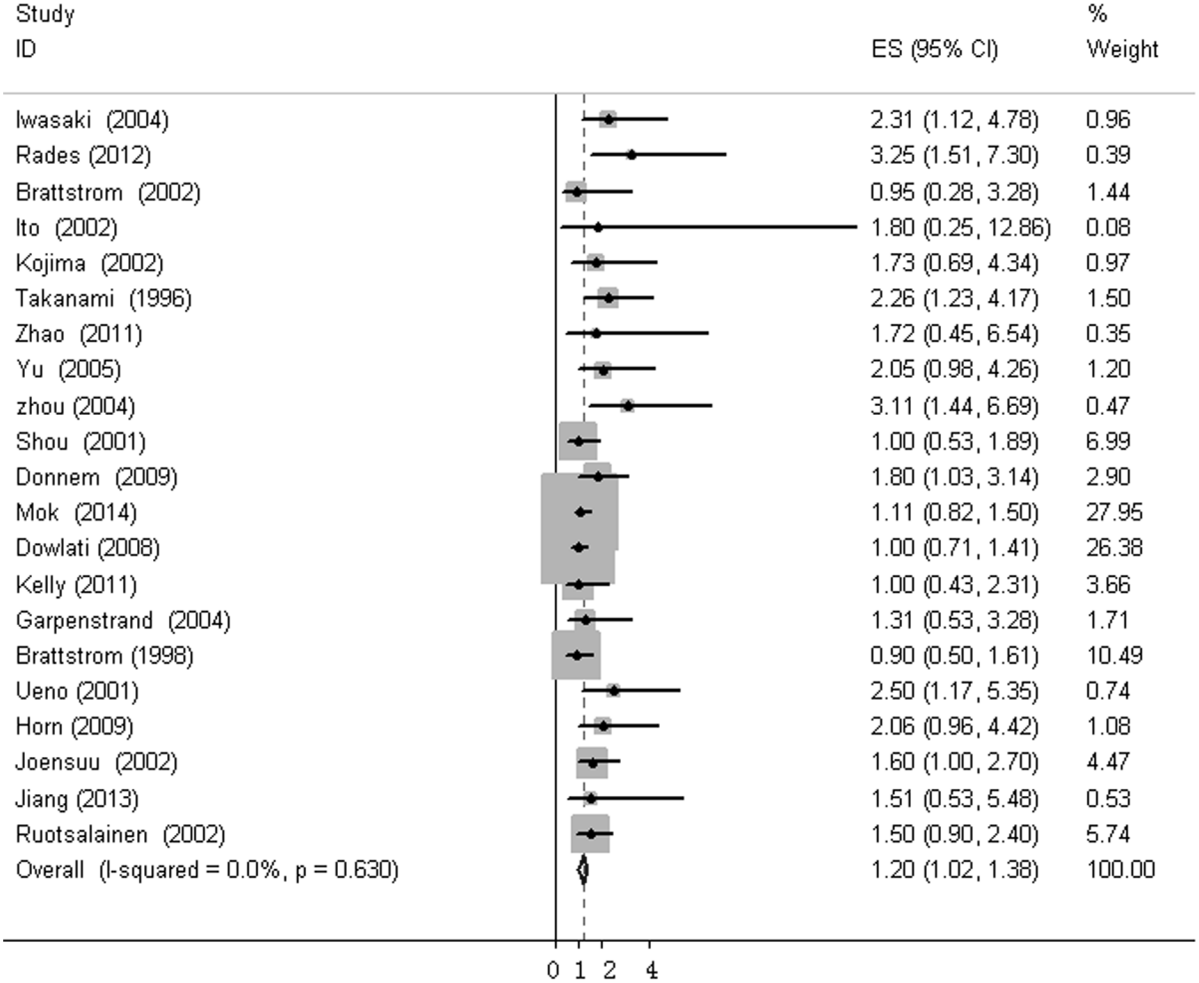
Forest plots of OS associated with bFGF expression in lung cancer.

**Fig 4:**
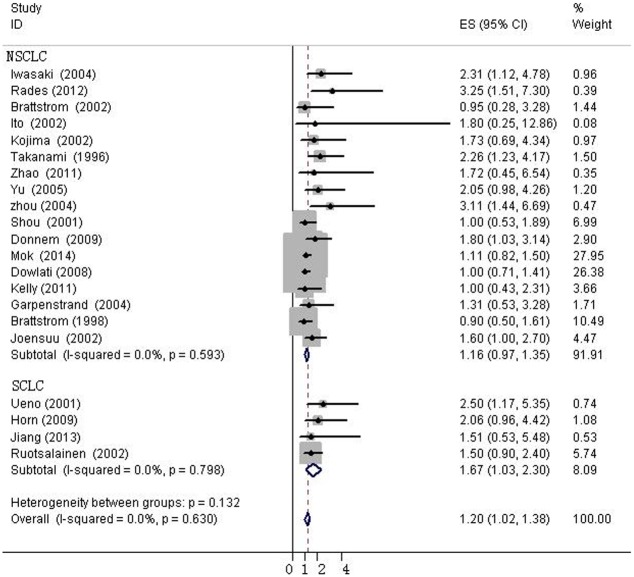
Forest plots of OS assessing bFGF expression in non-small cell lung cancer (NSCLC) and small cell lung cancer (SCLC).

**Fig 5:**
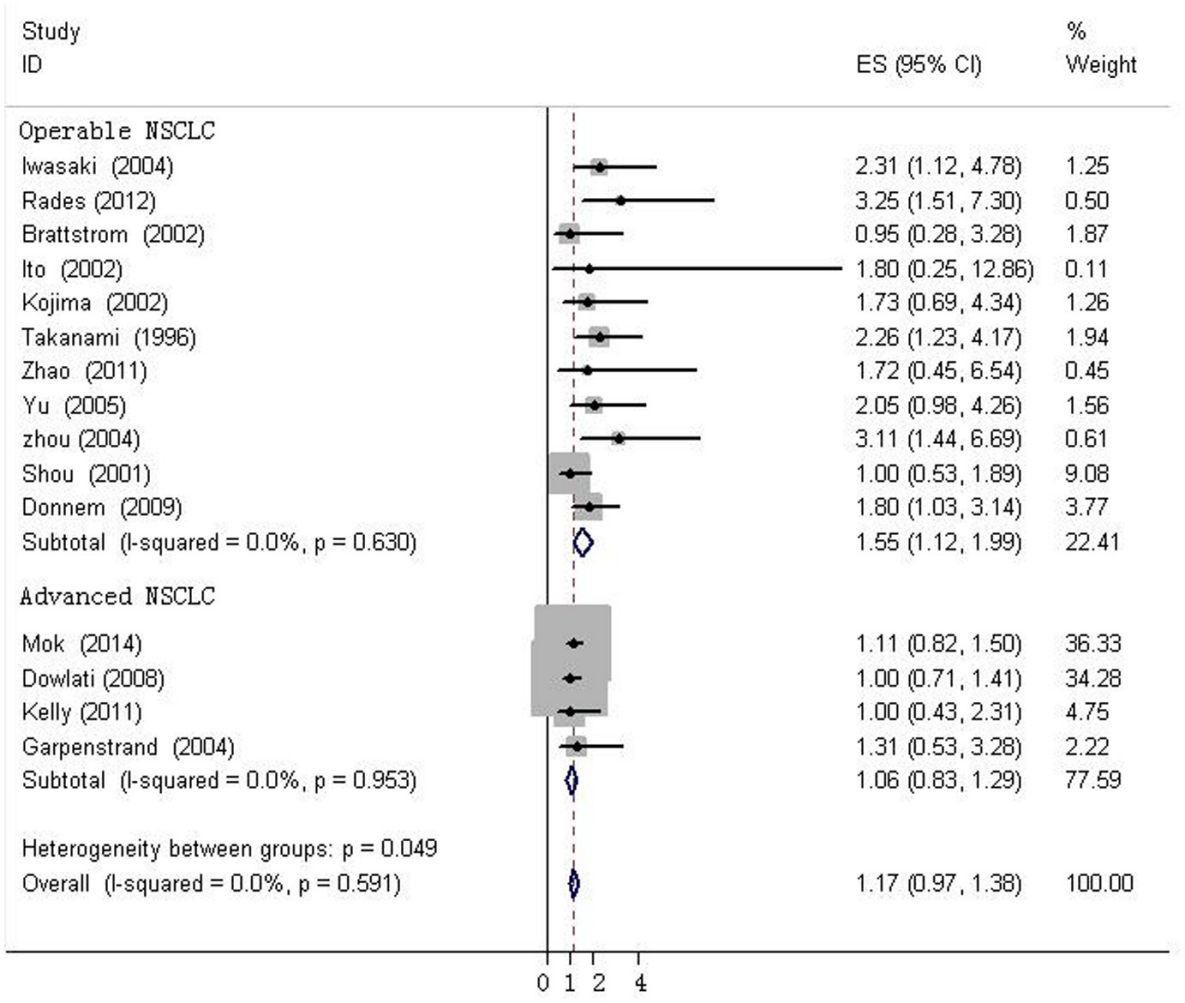
Forest plots of OS assessing bFGF expression in operable NSCLC advanced NSCLC.

**Fig 6:**
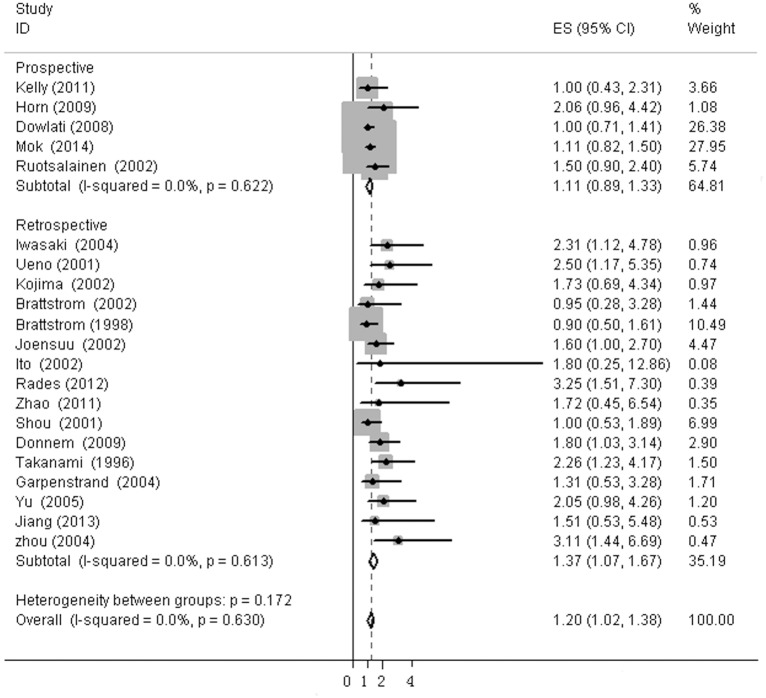
Forest plots of OS assessing bFGF expression in retrospective studies and prospective studies.

The heterogeneity of selected studies was examined according to the I^2^ statistic and p-value. Moderate heterogeneity was found in all of the eligible studies (I^2^ = 31.03%, p = 0.073) with a combined HR of 1.025 (95%CI, 0.872–1.179) as calculated by the random-effects model. Sensitivity analysis was conducted to explore heterogeneity (Fig 2). The IHC study by Behrens *et al*., which investigated bFGF expression in squamous carcinoma, was the main source of heterogeneity [31]. No heterogeneity was found among the other studies when the study by Behrens *et al*. was excluded (I^2^ = 0%, p = 0.630). The fixed-effects model was applied to calculate the HR of the remaining 21 studies. The combined HR evaluating bFGF overexpression on OS was 1.202 (95%CI, 1.022–1.382, Fig 3). The results showed that high bFGF expression was associated with poor OS in lung cancer. Moreover, subgroup analysis was performed according to the histological type (NSCLC versus SCLC). The HR for OS favored patients with low bFGF expression in SCLC (HR = 1.667, 95%CI, 1.035–2.299, I^2^ = 0%), while bFGF expression did not impact OS in the NSCLC group as a whole (HR = 1.16, 95%CI, 0.973–1.348, I^2^ = 0%, Fig 4). Additionally, there was no evidence showing heterogeneity in either group. In the subgroup of NSCLC, high bFGF expression indicated worse prognosis when the tumor was considered at an operable stage (HR = 1.553, 95%CI, 1.120–1.986, I^2^ = 0%), but it had no influence on advanced stage (HR = 1.060, 95%CI, 0.828–1.293, I^2^ = 0%, Fig 5). Furthermore, we conducted subgroup analysis according to study design. No heterogeneity was found in either group. The combined HR evaluating bFGF overexpression on OS in retrospective studies was 1.37 (95%CI 1.07–1.67). However, in prospective studies, bFGF overexpression did not show an impact on survival (HR = 1.11, 95%CI 0.89–1.33, Fig 6).

### Publication bias

Begg’s funnel plot was performed to assess publication bias in this meta-analysis. Eligible studies investigating patients with NSCLC in an operable stage and SCLC yielded a Begg’s test score of p = 0.938 and p = 0.849 respectively. No obvious publication bias was found in either the retrospective or prospective studies (p = 0.787 and p = 0.327 respectively, S2 File).

## Discussion

Emerging data on the bFGF signaling pathway has sparked several pharmaceutical companies to develop drugs that target bFGF and FGFRs. Efforts to develop anti-FGF or FGFR agents are also underway in cancer treatment [36], including a novel antiangiogenic bFGF antagonist, which could potentially block the activity of multiple FGF ligands and receptors, and exert both antiangiogenic and anti-proliferative effects [37]. Additionally, the FGF-FGFR pathway may function as a mechanism of resistance to anti-VEGF treatment. Dozens of clinical trials are being performed to treat various cancers with brivanib as monotherapy or in combination with other agents [38,39].

SCLC is initially chemosensitive, but rapidly relapses with chemoresistance, and has an OS of <5%. In recent years, several novel therapies have been developed for NSCLC, but not as many as in SCLC. Our analysis suggested that inhibiting bFGF-mediated angiogenesis also may be an effective treatment for SCLC. The FGFR inhibitor PD173074 blocks SCLC growth *in vitro* and *in vivo* [40]. BIBF1120 is a novel triple angiokinase inhibitor that predominantly blocks FGFR, VEGF and PDGFR. A phase II trial is ongoing to evaluate the efficacy of BIBF1120 in patients with recurrent SCLC.

In this meta-analysis, explicit criteria were set to screen eligible studies. Quality assessment was performed independently by two investigators according to the ELCWP scale designed for biological prognostic factors. Quantitative aggregation of the survival results was performed since there was no significant difference between significant and non-significant studies according tothe global score. Similarly, there was no difference in score between studies grouped by examining methods, patient populations or histological types.

We firstly combined 22 eligible studies and found a HR of 1.025 (95%CI, 0.872–1.179), indicating that OS was not associated with bFGF expression. However, there was moderate heterogeneity in the whole group (I^2^ = 31.03%, p = 0.073). One study that followed exclusively SCC patients was found to be the main source of heterogeneity through sensitivity analysis. The combined HR and 95%CI did not alter significantly when excluding any study but this one. We compared this article carefully with others, and we inferred that there may be two reasons for this heterogeneity. First, adenocarcinoma and SCC are not homogeneous, although both are categorized as NSCLC. In this paper, bFGF, FGFR1 and FGFR2 expression was examined in adenocarcinoma and SCC, and high bFGF expression was found to be a good predictor of OS in SCC. However, the effect of bFGF expression on adenocarcinoma was not mentioned (without any text description, data or chart). In the other reports, the histological type enrolled was NSCLC, non-SCC or adenocarcinoma. Additionally, the method of evaluating the IHC results in this article was different from that of the others. According to Behrens *et al*., cytoplasmic expression and nuclear expression were calculated by different methods. Nuclear overexpression of FGFR1 and FGFR2 significantly correlated with a worse outcome. In contrast, cytoplasmic overexpression of bFGF and FGFR2 significantly correlated with better OS. In the study by Zhao *et al*. [27], cytoplasmic and/or nuclear staining in the tumor cells was evaluated by the same criterion simultaneously. The remaining 8 articles used IHC to evaluate bFGF expression according to the cytoplasmic staining intensity of bFGF and/or the percentage of bFGF-positive tumor cells. Therefore, we excluded the Behrens study from the final analysis. There was no heterogeneity in the remaining 21 studies (I^2^ = 0%, p = 0.630) and the pooled HR was 1.202 (95%CI, 1.022–1.382), indicating a worse prognosis with high bFGF expression. However, in subgroups by histologic type and disease stage, bFGF expression remained significantly associated with OS in SCLC and operable NSCLC, but not in advanced NSCLC.

We also analyzed possible reasons for the difference between operable NSCLC and advanced NSCLC. Firstly, we observed that cells in the blood were analyzed in the four studies of advanced NSCLC, while in the ten reports on operable NSCLC, bFGF expression was evaluated in the tumor tissue. There are multiple possible sources of bFGF in the blood, such as platelets, mast cells, macrophages and cancer cells [41–44]. bFGF expression in the tumor is a more direct and robust indicator of the tumor burden than peripheral blood. Therefore, we thought that the prognostic impact of bFGF expression in blood versus tumor tissue might account for the difference in association among the stages of NSCLC. Secondly, angiogenesis is an important biological process and a relatively early event during lung cancer oncogenesis [45]. In advanced NSCLC, there are many more factors involved in angiogenesis than in early NSCLC. Therefore, we speculated that bFGF, as an important angiogenesis inducer, may exert more influence in early NSCLC than in advanced NSCLC. In 4 articles on SCLC, bFGF expression in the blood was examined and the combined HR is 1.667 (95%CI, 1.035–2.299, I^2^ = 0%). However, there were only 4 articles in the SCLC group, and each had a small sample size, so more well-designed studies are needed to draw conclusions in SCLC.

The identification of a molecular prognostic factor is necessary for improving the prediction of lung cancer prognosis; it may facilitate individualized risk-benefit assessment for treatment strategies. More intensive therapy may be necessary for patients with high bFGF expression for operable NSCLC and SCLC. A reliable identification of an angiogenic-related factor would be desirable, not only for risk assessment, but also for the implementation of angiogenesis targeted treatment. Currently, several bFGF-FGFR pathway inhibitors are undergoing clinical trials, particularly in NSCLC. These agents targeting the FGF signaling pathway, in combination with adjuvant or neoadjvant therapy, could potentially prolong the survival of NSCLC patients undergoing curative surgical resection.

Begg’s funnel plot indicated that publication bias was not present in our analysis. However, publication bias could not be avoided completely: we limited our analysis to articles published in English or Chinese, which probably introduced bias, and there might have been a publication bias for positive over negative results.

However, our study has several limitations, as we could not prevent all potential bias among these studies. Although 21 studies were included in the final analysis, the average sample size was small (mean,98). For articles without HR and 95%CI, we contacted the corresponding author, but received no reply. Consequently, we extrapolated these metrics from the data or curves in these articles indirectly, which could be another potential source of bias. Different methods of examining bFGF expression, primary antibody source and concentration, and threshold cut-off values may introduce more bias as well. IHC is a relatively complicated technique with many steps and is observer-dependent. Moreover, our meta-analysis data did not include information on age, smoking status, tumor size and other factors, which might result in confounding bias. Lastly, we included studies which dichotomized bFGF expression into the high and low expression group. Other studies that included bFGF expression as a continuous variable were excluded because the data was not extractable.

Despite the above limitations, our meta-analysis revealed that bFGF overexpression has significant impact on survival in lung cancer patients. For operable NSCLC and SCLC patients, bFGF overexpression is an unfavorable prognostic factor and could be helpful in optimizing therapeutic schemes. However, more studies need to be carried out to investigate the prognostic value of bFGF to other kinds of cancer.

## Supporting Information

S1 File PRISMA 2009 Checklist. (TIF)

S2 File Begg’s funnel plot for studies included in this meta-analysis. Funnel plot for publication bias of OS in operable non-small cell lung cancer (NSCLC)(Figure A in S2 File). Funnel plot for publication bias test of OS in small cell lung cancer (SCLC)(Figure B in S2 File). Funnel plot for publication bias test of OS in retrospective studies(Figure C in S2 File). Funnel plot for publication bias test of OS in prospective studies(Figure D in S2 File).
(TIF)

## References

[1] McMillen E, Ye F, Li G, Wu Y, Yin G, Liu W. Epidermal growth factor receptor (EGFR) mutation and p-EGFR expression in resected non-small cell lung cancer. Exp Lung Res 2010;36:531–7. 10.3109/01902148.2010.482176 20939760

[2] Wao H, Mhaskar R, Kumar A, Miladinovic B, Djulbegovic B. Survival of patients with non-small cell lung cancer without treatment: a systematic review and meta-analysis. Syst Rev. 2013; 4:2–10. 10.1186/2046-4053-2-10 PMC3579762 23379753

[3] Folkman J. What is the evidence that tumors are angiogenesis dependent? J Natl Cancer Inst 1990;82:4–6. 1688381 10.1093/jnci/82.1.4

[4] Cao Y, Linden P, Farnebo J, Cao R, Eriksson A, Kumar V, et al Vascular endothelial growth factor C induces angiogenesis in vivo. Proc Natl Acad Sci U S A 1998;95:14389–94. 9826710 10.1073/pnas.95.24.14389 PMC24383

[5] Beenken A, Mohammadi M. The FGF family: biology, pathophysiology and therapy. Nat Rev Drug Discov 2009;8:235–53. 10.1038/nrd2792 19247306 PMC3684054

[6] Turner N, Grose R. Fibroblast growth factor signalling: from development to cancer. Nat Rev Cancer 2010;10:116–29. 10.1038/nrc2780 20094046

[7] Okada-Ban M, Thiery JP, Jouanneau J. Fibroblast growth factor-2. Int J Biochem Cell Biol 2000;32:263–7. 10716624 10.1016/s1357-2725(99)00133-8

[8] Eto H, Suga H, Aoi N, Kato H, doi K, Kuno S, et al Therapeutic potential of fibroblast growth factor-2 for hypertrophic scars: upregulation of MMP-1 and HGF expression. Lab Invest 2012;92:214–23. 10.1038/labinvest.2011.127 21946856

[9] Kim HR, Heo YM, Jeong KI, Kim YM, Jang HL, LEE KY, et al FGF-2 inhibits TNF-α mediated apoptosis through upregulation of Bcl2-A1 and Bcl-xL in ATDC5 cells. BMB Rep 2012;45:287–92. 22617452 10.5483/bmbrep.2012.45.5.287

[10] Xiao D, Wang K, Zhou J, Cao H, Deng Z, Hu Y, et al Inhibition of fibroblast growth factor 2-induced apoptosis involves survivin expression, protein kinase C alpha activation and subcellular translocation of Smac in human small cell lung cancer cells. Acta Biochim Biophys Sin (Shanghai) 2008;40:297–303. 18401527 10.1111/j.1745-7270.2008.00401.x

[11] Nishida T, Kubota S, Aoyama E, Janune D, Maeda A, Takigawa M. Effect of CCN2 on FGF2-induced proliferation and MMP9 and MMP13 productions by chondrocytes. Endocrinology 2011;152:4232–41. 10.1210/en.2011-0234 21914781

[12] Steels E, Paesmans M, Berghmans T, Branle F, Lemaitre F, Mascaux C, et al Role of p53 as a prognostic factor for survival in lung cancer: a systematic review of the literature with a meta-analysis. Eur Respir J 2001;18:705–19. 11716177 10.1183/09031936.01.00062201

[13] Tierney JF, Stewart LA, Ghersi D, Burdett S, Sydes MR. Practical methods for incorporating summary time-to-event data into meta-analysis. Trials 2007;8:16 17555582 10.1186/1745-6215-8-16 PMC1920534

[14] Takanami I, Tanaka F, Hashizume T, Kikuchi K, Yamanoto Y, Yamanoto T, et al The basic fibroblast growth factor and its receptor in pulmonary adenocarcinomas: an investigation of their expression as prognostic markers. Eur J Cancer 1996; 32A:1504–9. 8911109 10.1016/0959-8049(95)00620-6

[15] Ito H, Oshita F, Kameda Y, Suzuki R, Ikehara M, Aria H, et al Expression of vascular endothelial growth factor and basic fibroblast growth factor in small adenocarcinomas. Oncol Rep 2002;9:119–23. 11748468

[16] Kojima H, Shijubo N, Abe S. Thymidine phosphorylase and vascular endothelial growth factor in patients with Stage I lung adenocarcinoma.Cancer 2002 2 15;94(4):1083–93. 11920479 10.1002/cncr.10352

[17] Mok T, Gorbunova V, Juhasz E, Szima B, Burdaeva O, Orlov S, et al A correlative biomarker analysis of the combination of bevacizumab and carboplatin-based chemotherapy for advanced nonsquamous non-small-cell lung cancer: results of the phase II randomized ABIGAIL study (BO21015). J Thorac Oncol 2014;9:848–55. 24807156 10.1097/JTO.0000000000000160

[18] Dowlati A, Gray R, Sandler AB, Schiller JH, Johnson DH. Cell adhesion molecules, vascular endothelial growth factor, and basic fibroblast growth factor in patients with non-small cell lung cancer treated with chemotherapy with or without bevacizumab—an Eastern Cooperative Oncology Group Study. Clin Cancer Res. 2008;14:1407–12. 10.1158/1078-0432.CCR-07-1154 18316562

[19] Brattström D, Bergqvist M, Larsson A, Holmertz J, Hesselius P, Rosenberg L, et al Basic fibroblast growth factor and vascular endothelial growth factor in sera from non-small cell lung cancer patients. Anticancer Res 1998;18:1123–7. 9615776

[20] Joensuu H, Anttonen A, Eriksson M, Mäkitaro R, Alfthan H, Kinnula V, et al Soluble syndecan-1 and serum basic fibroblast growth factor are new prognostic factors in lung cancer. Cancer Res 2002;62:5210–7. 12234986

[21] Garpenstrand H, Bergqvist M, Brattström D, Larsson A, Oreland L, Hesselius P, et al Serum semicarbazide-sensitive amine oxidase (SSAO) activity correlates with VEGF in non-small-cell lung cancer patients. Med Oncol 2004;21:241–50. 15456951 10.1385/MO:21:3:241

[22] Iwasaki A, Kuwahara M, Yoshinaga Y, Shirakusa T. Basic fibroblast growth factor (bFGF) and vascular endothelial growth factor (VEGF) levels, as prognostic indicators in NSCLC. Eur J Cardiothorac Surg 2004;25:443–8. 15019676 10.1016/j.ejcts.2003.11.031

[23] Zhou T, Pan TC. Correlation of angiogenesis with expression of basic fibroblast growth factor and its receptor in lung cancer. Academic Dissertation, Tongji Medical College, Huazhong University of Science and Technology, 2004.

[24] Yu M, Li SY, Yu Z, Qiu XS, Hou P, Wang EH, et al Clinical significance of heparanase and basic fibroblast growth factor expression in human non-small cell lung cancer. Chinese Journal of Pathology 2005;34:36–41. 15796880

[25] Donnem T, Al-Shibli K, Al-Saad S, Busund LT, Bremnes RM. Prognostic impact of fibroblast growth factor 2 in non-small cell lung cancer: coexpression with VEGFR-3 and PDGF-B predicts poor survival. J Thorac Oncol 2009; 4:578–85. 10.1097/JTO.0b013e31819f2e38 19318994

[26] Brattström D, Bergqvist M, Hesselius P, Larsson A, lamberg K, Wernlund J, et al Elevated preoperative serum levels of angiogenic cytokines correlate to larger primary tumours and poorer survival in non-small cell lung cancer patients. Lung Cancer 2002;37:57–63. 12057868 10.1016/s0169-5002(02)00027-2

[27] Zhao M, Gao FH, Wang JY, Liu F, Yuan HH, Zhang WY, et al JAK2/STAT3 signaling pathway activation mediates tumor angiogenesis by upregulation of VEGF and bFGF in non-small-cell lung cancer. Lung Cancer. 2011;73:366–74. 10.1016/j.lungcan.2011.01.002 21333372

[28] Shou Y, Hirano T, Gong Y, Kato Y, Yoshida K, Ohira T, et al Influence of angiogenetic factors and matrix metalloproteinases upon tumour progression in non-small-cell lung cancer. Br J Cancer. 2001;85:1706–12. 11742492 10.1054/bjoc.2001.2137 PMC2363988

[29] Kelly RJ, Rajan A, Force J, Lopez-Chavez A, Keen C, Cao L, et al Evaluation of KRAS mutations, angiogenic biomarkers, and DCE-MRI in patients with advanced non-small-cell lung cancer receiving sorafenib. Clin Cancer Res 2011;17:1190–9. 10.1158/1078-0432.CCR-10-2331 21224376 PMC3048919

[30] Rades D, Setter C, Dahl O, Schild SE, Noack F. Fibroblast growth factor 2—a predictor of outcome for patients irradiated for stage II-III non-small-cell lung cancer. Int J Radiat Oncol Biol Phys 2012;82:442–7. 10.1016/j.ijrobp.2010.08.048 20950963

[31] Behrens C, Lin HY, Lee JJ, Raso MG, Hong WK, Wistuba II, et al Immunohistochemical expression of basic fibroblast growth factor and fibroblast growth factor receptors 1 and 2 in the pathogenesis of lung cancer. Clin Cancer Res 2008; 14:6014–22. 10.1158/1078-0432.CCR-08-0167 18829480 PMC5108626

[32] Ueno K, Inoue Y, Kawaguchi T, Hosoe S, Kawahara M. Increased serum levels of basic fibroblast growth factor in lung cancer patients: relevance to response of therapy and prognosis. Lung Cancer 2001;31:213–9. 11165400 10.1016/s0169-5002(00)00187-2

[33] Ruotsalainen T, Joensuu H, Mattson K, Salven P. High pretreatment serum concentration of basic fibroblast growth factor is a predictor of poor prognosis in small cell lung cancer. Cancer Epidemiol Biomarkers Prev 2002; 11:1492–5. 12433733

[34] Jiang W, Cao JY, Pan B, Yu Y. Clinical significance of serum vascular endothelial growth factor and b-fibroblast growth factor before and after chemotherapy in patients with small cell lung cancer. Chinese Journal of Clinical Oncology 2013;40:638–642.

[35] Horn L, Dahlberg SE, Sandler AB, Dowlati A, Moore DF, Murren JR, et al Phase II study of cisplatin plus etoposide and bevacizumab for previously untreated, extensive-stage small-cell lung cancer: Eastern Cooperative Oncology Group Study E3501. J Clin Oncol 2009;27:6006–11. 10.1200/JCO.2009.23.7545 19826110 PMC2793043

[36] Turner N, Grose R. Fibroblast growth factor signalling: from development to cancer. Nat Rev Cancer 2010;10:116–29. 10.1038/nrc2780 20094046

[37] Alessi P, Leali D, Camozzi M, Cantelmo A, Albini A, Presta M. Anti-FGF2 approaches as a strategy to compensate resistance to anti-VEGF therapy: long-pentraxin 3 as a novel antiangiogenic FGF2-antagonist. Eur Cytokine Netw 2009;20:225–34. 10.1684/ecn.2009.0175 20167562

[38] Gyanchandani R, Ortega Alves MV, Myers JN, Kim S. A proangiogenic signature is revealed in FGF-mediated bevacizumab-resistant head and neck squamous cell carcinoma. Mol Cancer Res. 2013; 11:1585–96. 10.1158/1541-7786.MCR-13-0358 24092775 PMC3955724

[39] Semrad TJ, Mack PC. Fibroblast growth factor signaling in non-small-cell lung cancer. Clin Lung Cancer. 2012; 13:90–5. 10.1016/j.cllc.2011.08.001 21959109

[40] Pardo OE, Latigo J, Jeffery RE, Nye E, Poulsom R, Spencer-Dene B, et al The fibroblast growth factor receptor inhibitor PD173074 blocks small cell lung cancer growth in vitro and in vivo. Cancer Res 2009;69:8645–51. 10.1158/0008-5472.CAN-09-1576 19903855

[41] Middleton KK, Barro V, Muller B, Terada S, Fu FH. Evaluation of the effects of platelet-rich plasma (PRP) therapy involved in the healing of sports-related soft tissue injuries. Iowa Orthop J. 2012;32:150–63. 23576936 PMC3565396

[42] Dyduch G, Kaczmarczyk K, Okoń K. Mast cells and cancer: enemies or allies? Pol J Pathol. 2012;63:1–7. 22535614

[43] Riabov V, Gudima A, Wang N, Mickley A, Orekhov A, Kzhyshkowska J. Role of tumor associated macrophages in tumor angiogenesis and lymphangiogenesis. Front Physiol. 2014;5:75 10.3389/fphys.2014.00075 24634660 PMC3942647

[44] Colman RW, Wu Y, Liu Y. Mechanisms by which cleaved kininogen inhibits endothelial cell differentiation and signalling. Thromb Haemost. 2010;104:875–85. 10.1160/TH10-01-0017 20886177

[45] Farhat FS, Tfayli A, Fakhruddin N, Mahfouz R, Otrock ZK, Alameddine RS, et al Expression, prognostic and predictive impact of VEGF and bFGF in non-small cell lung cancer. Crit Rev Oncol Hematol. 2012;84:149–60. 10.1016/j.critrevonc.2012.02.012 22494932

