# Supplementary figures and images for: Prognostic Value of Basic Fibroblast Growth Factor (bFGF) in Lung Cancer: A Systematic Review with Meta-Analysis
Source: PLoS One. 2016 Jan 29;11(1):e0147374. doi: 10.1371/journal.pone.0147374 (PMC4732945; doi:10.1371/journal.pone.0147374)

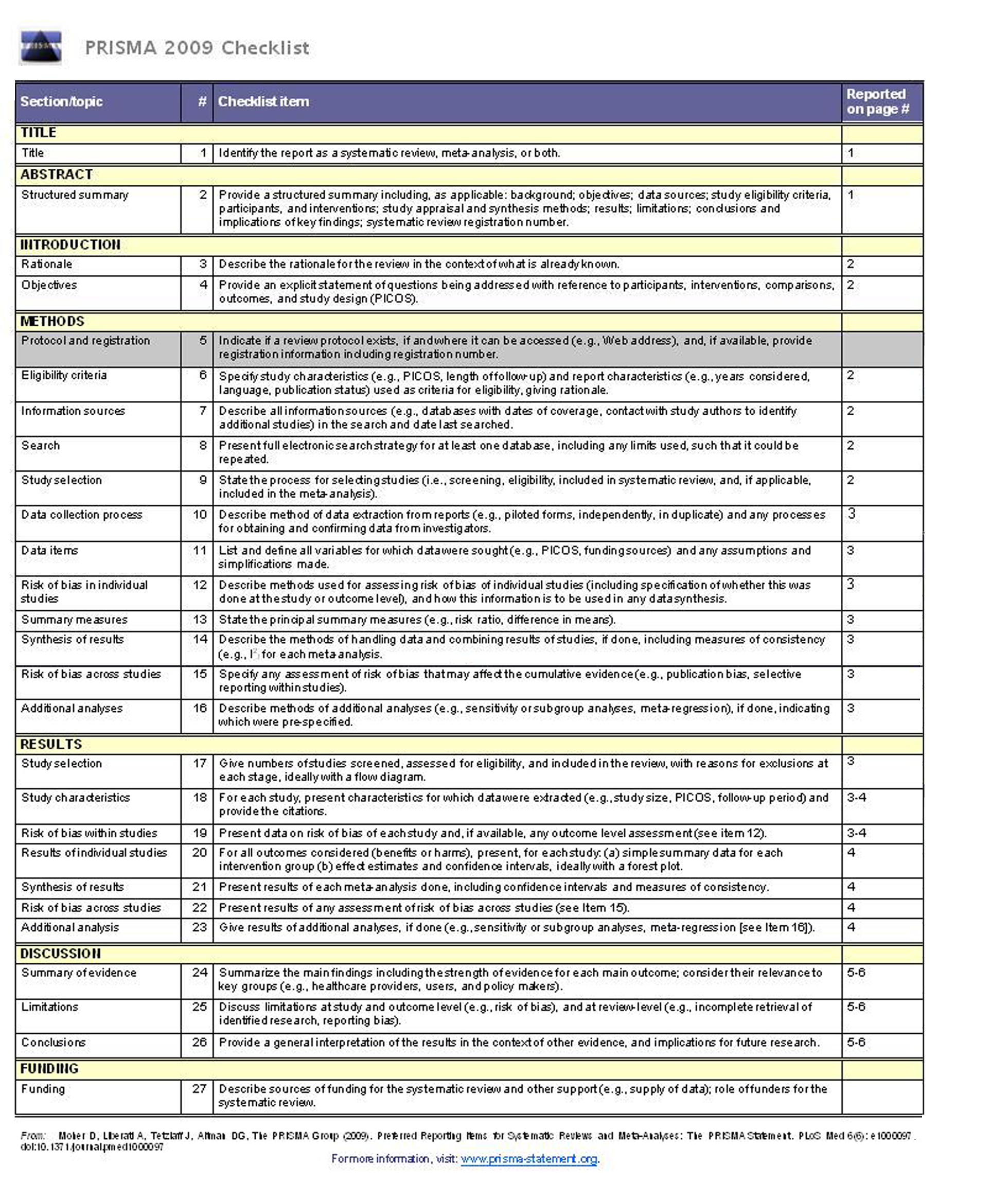

Supplement: S1 File — (TIF) [file pone.0147374.s001.tif]

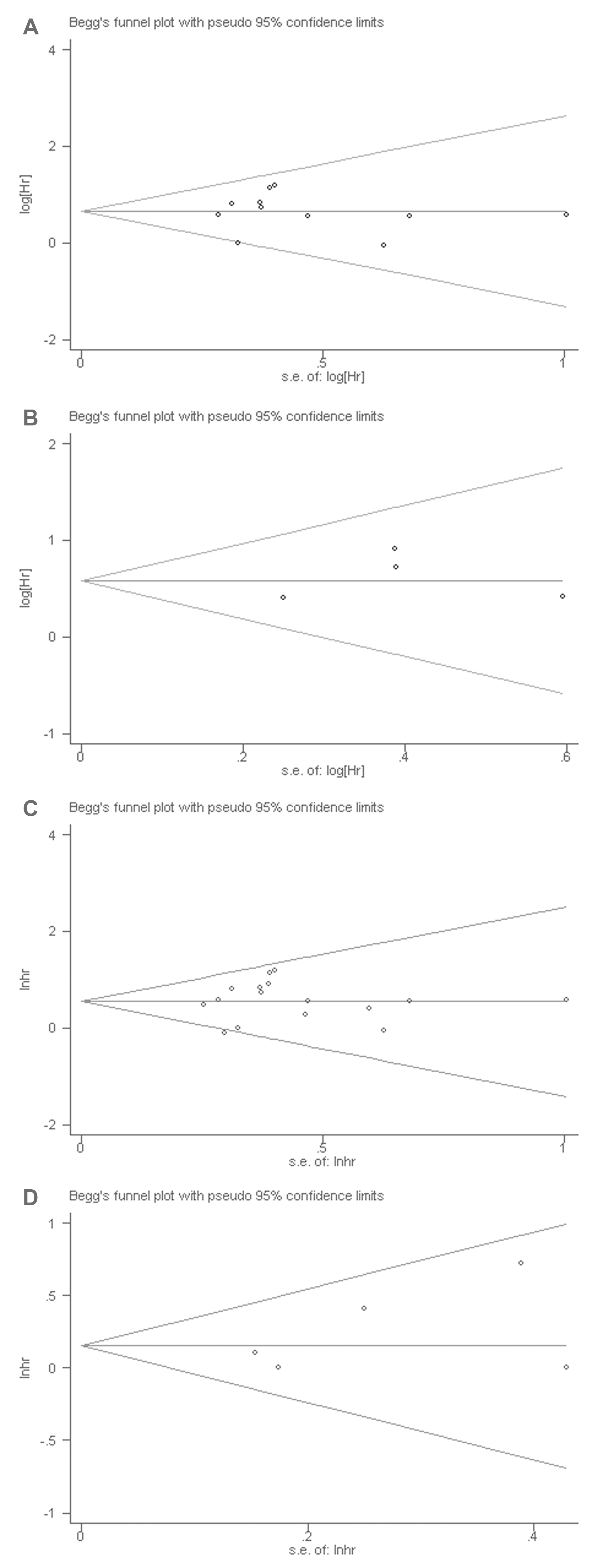

Supplement: S2 File — Funnel plot for publication bias of OS in operable non-small cell lung cancer (NSCLC)(Figure A in S2 File). Funnel plot for publication bias test of OS in small cell lung cancer (SCLC)(Figure B in S2 File). Funnel plot for publication bias test of OS in retrospective studies(Figure C in S2 File). Funnel plot for publication bias test of OS in prospective studies(Figure D in S2 File). (TIF) [file pone.0147374.s002.tif]
